# Robot-assisted gait training (Lokomat) improves walking function and activity in people with spinal cord injury: a systematic review

**DOI:** 10.1186/s12984-017-0232-3

**Published:** 2017-03-23

**Authors:** Ki Yeun Nam, Hyun Jung Kim, Bum Sun Kwon, Jin-Woo Park, Ho Jun Lee, Aeri Yoo

**Affiliations:** 1 Department of Physical Medicine & Rehabilitation, Dongguk University College of Medicine, Goyang, Korea; 2 Department of Preventive Medicine, Korea University College of Medicine, Seoul, Korea; 3 Central Seoul Eye Center, Seoul, South Korea

**Keywords:** Spinal Cord Injuries, Gait, Robotics, Locomotion, Physical therapy

## Abstract

**Abstract:**

Robot-assisted gait training (RAGT) after spinal cord injury (SCI) induces several different neurophysiological mechanisms to restore walking ability, including the activation of central pattern generators, task-specific stepping practice and massed exercise. However, there is no clear evidence for the optimal timing and efficacy of RAGT in people with SCI. The aim of our study was to assess the effects of RAGT on improvement in walking-related functional outcomes in patients with incomplete SCI compared with other rehabilitation modalities according to time elapsed since injury. This review included 10 trials involving 502 participants to meta-analysis. The acute RAGT groups showed significantly greater improvements in gait distance, leg strength, and functional level of mobility and independence than the over-ground training (OGT) groups. The pooled mean difference was 45.05 m (95% CI 13.81 to 76.29, P = 0.005, I^2^ = 0%; two trials, 122 participants), 2.54 (LEMS, 95% CI 0.11 to 4.96, P = 0.04, I^2^ = 0%; three trials, 211 participants) and 0.5 (WISCI-II and FIM-L, 95% CI 0.02 to 0.98, P = 0.04, I^2^ = 67%; three trials, 211 participants), respectively. In the chronic RAGT group, significantly greater improvements in speed (pooled mean difference = 0.07 m/s, 95% CI 0.01 to 0.12, P = 0.01, I^2^ = 0%; three trials, 124 participants) and balance measured by TUG (pooled mean difference = 9.25, 95% CI 2.76 to 15.73, P = 0.005, I^2^ = 74%; three trials, 120 participants) were observed than in the group with no intervention. Thus, RAGT improves mobility-related outcomes to a greater degree than conventional OGT for patients with incomplete SCI, particularly during the acute stage. RAGT treatment is a promising technique to restore functional walking and improve locomotor ability, which might enable SCI patients to maintain a healthy lifestyle and increase their level of physical activity.

**Trial registration:**

PROSPERO (CRD 42016037366). Registered 6 April 2016.

## Background

A spinal cord injury (SCI) is a lesion of neural elements of the spinal cord, it is a devastating condition with a major impact on a person’s life. Locomotor ability is frequently affected in people with SCI, and decreased mobility after SCI is associated with a heightened risk of a decrease in both life satisfaction and quality of life [1]. Locomotor training focuses on retraining the motor function via plastic change [2], and the neurophysiological mechanism underlying the restoration of human locomotion after SCI involve enhancing the afferent input to the spinal cord and activating central pattern generators (CPG) embedded within the lumbosacral spinal cord. Plastic changes can be induced in both the spinal cord level and sensory motor cortex via intensive locomotor training, but only in incomplete SCI subjects [3]. Motor recovery in SCI patients can be improved with both conventional overground walking training (OGT) and body weight-supported (BWS) treadmill training. BWS treadmill training enables early initiation of gait training, integration of weight-bearing activities, stepping and balance using a task-specific approach and symmetrical gait pattern [4]. To replicate a normal gait pattern during manually facilitated BWS treadmill training, two or three therapists are needed to control and assist with trunk and limb kinematics.

Manual training is strenuous and exhausting for therapists, so sophisticated automated electromechanical devices have been developed [5]. In the late 1990s, robot-assisted gait training (RAGT) was introduced. It offers several advantages, including the ability to increase the intensity and total duration of training while maintaining a physiological gait pattern. Also, the task-specific stepping practice is known to enhance the afferent feedback associated with normal locomotion and can induce plasticity in the involved motor centers [6, 7]. Moreover, locomotor robotic devices can reduce personnel costs involved in manual assistance training, which can require up to three physical therapists.

Several studies have evaluated RAGT in incomplete SCI patients. Although some of the results were encouraging, there is still uncertainty regarding the appropriate timing of RAGT following SCI, and no clear evidence of the efficacy of RAGT in terms of SCI patients’ gait functional outcomes, such as walking ability (i.e., gait speed and distance), body functions (i.e., lower extremity motor score and spasticity), and functional level of mobility and independence has been shown. The aim of the present study was to assess the effects of RAGT on improving walking-related functional outcomes according to time since injury in patients with incomplete SCI, as measured relative to other rehabilitation modalities.

## Method

### Materials and methods

We used comprehensive databases to find studies comparing RAGT with any other exercise or physiotherapy. This study was performed according to the Cochrane Review Methods and reported according to the Preferred Reporting Items for Systematic Reviews and Meta-Analyses statement [8].

### Data source & literature source

Randomized trials were identified by searching MEDLINE, EMBASE, SCOPUS, Web of Science, Cochrane Central Register of Controlled Trials, the World Health Organization International Clinical Trials Registry Platform, and the clinical trials registry and database of the U.S. National Institutes of Health (ClinicalTrials.gov) on January 17 2016. We put no restrictions on language or year of publication in our search. The following keywords were searched: spinal cord injuries, gait disorders, neurologic, and robotics. See *Appendix* 1 for a comprehensive list (MEDLINE, EMBASE, CENTRAL Web of Science and Scopus). Search strategies were developed for each database using both free-text terms and the controlled vocabulary (MeSH and Emtree). We also searched the reference lists of included studies and other reviews to identify additional trials. Duplicate records were identified by title, authors and journal citations and removed.

### Study selection

Study inclusion was decided independently by two reviewers (ARY and KYN) based on the selection criteria. Studies were selected in two stages, as follows: First, we screened the titles and abstracts of identified studies. Second, we screened the full text. We included randomized controlled trials (RCTs) of parallel-group or cross-over design involving patients with SCI. Studies were included in our meta-analysis if they compared RAGT to a control comprising any other exercise or no treatment; or involved participants with an incomplete, traumatic or nontraumatic, nonprogressive SCI, as defined by AIS grades B, C, or D; [9] participants were a minimum of 16 years of age because most neurologic development is complete once adolescence is reached; [10] training parameters were specified in detail; and locomotor or locomotor-related outcomes were evaluated.

### Data extraction

The two reviewers independently extracted data from each study using a predefined data extraction form. Disagreements were resolved through discussion or, if required, adjudication by a third reviewer.

The following variables were extracted from studies: (1) mean and SD of walking speed, walking capacity, walking independence and safety and incidence of adverse events during the trial in the intervention and control groups; (2) demographic, clinical, and treatment characteristics (e.g., number of patients in the intervention and control groups); (3) intervention and control protocol type; and (4) method of assessment. If the above variables were not mentioned in the studies, the data were requested from the authors via email.

### Assessment of methodological quality

The quality of included trials was assessed by extracting PEDro Scale scores from the Physiotherapy Evidence Database (www.pedro.org.au). The PEDro Scale has 11 items and is designed to rate the methodological quality (internal validity and statistical information) of randomized trials. Each item, with the exception of item #1, contributes one point to the total PEDro score (range, 0 to 10 points). The PEDro score is a valid measure of the internal validity and completeness of reporting. It has undergone Rasch analysis and showed moderate levels of inter-rater reliability (ICC 0.68, 95% CI 0.57 to 0.76) [11, 12]. Trials scoring < 6 were deemed to be of low quality [13]. Tests for funnel plot asymmetry are generally performed only when at least 10 studies are included in a meta-analysis [14]. Although 10 studies were included in this analysis, when sorted by outcomes, each outcome contained fewer than 9 studies. Thus publication bias in these trials could not be assessed.

### Statistical analysis

The main outcome was ambulatory function measured as the speed (m/s) and capacity (2 and 6 min gait distance) of walking. Other frequently investigated outcomes were the lower extremities motor scale (LEMS), functional independence measure – locomotion (FIM-L), walking index for spinal cord injury (WISCI), modified Ashworth scale (MAS), and timed up and go (TUG) test. We used weighted mean differences to estimate the treatment effects for ambulatory function. The weighted mean difference and 95% confidence intervals (CI) were calculated using the inverse variance method with random-effects weighting. We pooled the data as change values for all outcomes, if available. If not, they were estimated from the final and baseline values.

The statistical heterogeneity between the studies was evaluated by Cochran’s Q test and quantified with the I^2^ statistic (I^2^ ≥ 50% indicated substantial (moderate, high) heterogeneity) [15]. To identify the sources of heterogeneity, subgroup analyses were conducted according to (1) type of control [no intervention, body weight-supported gait training (BWS), OGT, and strength exercise], (2) time since injury [acute (<6 months), chronic (>12 months), and unknown]. We used RevMan version 5.2 for these analyses.

## Results

### Identification of studies

**Fig. 1:**
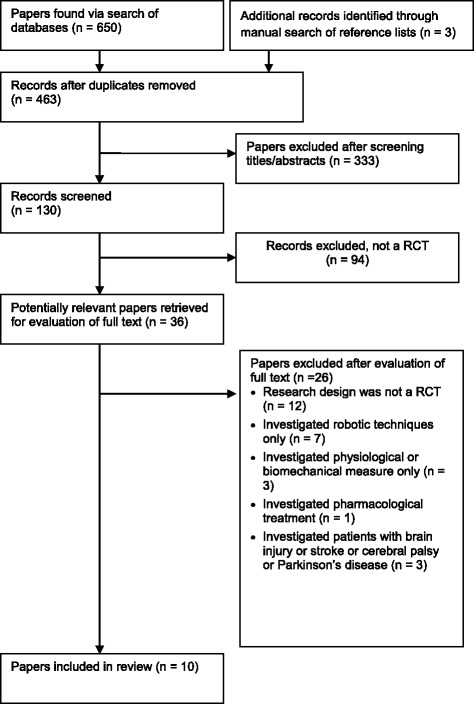
Flow of studies through the review

Searches of the databases resulted in identification of 653 articles (Fig. 1). Of these, 333 publications were excluded as they did not fulfill the selection criteria. For the remaining 130 articles, we obtained full manuscripts, and following scrutiny of these, we identified 36 potentially relevant studies and 26 publications were excluded as they were:

- 12 studies were not a RCTs
- 3 studies investigated physiological or biomechanical measures only
- 1 study investigated pharmacological treatment
- 7 studies investigated robotic techniques only (These papers were comparative studies of the operation of RAGT devices.)
- 3 studies investigated patients with brain injury, stroke, cerebral palsy, or Parkinson’s disease

Therefore, 10 studies were included in the review. Five trials were conducted in the U.S. [16–20], 2 in Spain [21, 22], 1 in Switzerland [23], 1 in China [24], and 1 in Korea. [25] All trials compared parallel intervention groups, and one study [23] used a crossover design. All studies were published in English. Two articles [16, 26] were based on the same trial, so the participants were counted only once. Another one of the reports [17] replaces and completes preliminary study results [27].

### Study characteristics and patient populations

#### Participants

The demographic characteristics of all 502 participants in the 10 studies are shown in Table 2. The number of participants in each study ranged from 9 [8, 23] to 88 [22] and the age of the participants ranged from 16 [22] to 70 years [22, 23]; more males than females participated. All included studies provided information on the level of spinal cord injury (C2 to L3) and baseline severity (AIS grades B to D); i.e., incomplete SCI. Most studies involved upper motor neuron lesions only, but two [22, 24] also included participants with lower motor neuron lesions. Most studies were AIS grade C/D [16, 17, 20–23] or D [24, 25], motor incomplete SCI only, but two studies [18, 19] included AIS grade B/C/D, motor or sensory incomplete SCI. Of the participants, 263 in four studies [18, 21, 22, 25] were assessed at < 6 months post-injury and 209 in five studies [16, 17, 19, 20, 23] were assessed at > 12 months post-injury. The remaining 30 participants in one study (mean 6.3 months post-injury) did not belong to any group [24]. To account for possible spontaneous neurologic recovery, any trials involving participants at an early or acute stage after SCI (<1 year post-injury) [21, 28–31] and those whose participants were at a chronic stage (>1 year post-injury) [17, 27, 31] were analyzed separately [2, 32, 33]. Acute participants in all studies were seen at ≤6 months since injury.

#### Quality

The mean PEDro score of the studies was 5.7 (range, 3 to 8) (Table 1). Two trials [21, 22] scored 8 on the PEDro scale, which was the highest possible score given the nature of the intervention since it would not be feasible to blind clinicians or participants. The majority of the studies were randomized (100%), analyzed between-group differences (100%), reported point estimates and variability (90%), had similar groups at baseline (50%), reported < 15% loss to follow-up (90%) and had blinded assessors (50%). The majority of the studies did not conceal the allocation list (70%), carry out an intention-to-treat analysis (60%), or blind participants or therapists (100%).

**Table 1:** PEDro criteria and scores of included studies (*n*=10)

Study	Random allocation	Concealed allocation	Groups similar at baseline	Participant blinding	Therapist blinding	Assessor blinding	<15% dropouts	Intention-to-treat analysis	Between-group difference reported	Point estimate and variability reported	Total (0 to 10)
Alcobendas-Maestro 2012	Y	Y	Y	N	N	Y	Y	Y	Y	Y	8
Duffell 2015	Y	N	Y	N	N	N	N	N	Y	Y	4
Esclarin-Ruz 2014	Y	Y	Y	N	N	Y	Y	Y	Y	Y	8
Field-Fote 2011	Y	N	Y	N	N	Y	Y	N	Y	Y	6
Hornby 2005	Y	N	N	N	N	N	Y	N	Y	N	3
Labruyere 2014	Y	N	N	N	N	Y	Y	Y	Y	Y	6
Niu 2014	Y	N	N	N	N	N	Y	Y	Y	Y	5
Shin 2014	Y	N	Y	N	N	N	Y	N	Y	Y	5
Tang 2014	Y	N	N	N	N	N	Y	Y	Y	Y	5
Varoqui 2014	Y	Y	N	N	N	Y	Y	Y	Y	Y	7

#### Interventions

All studies investigated the robotic-assisted device ‘Lokomat’ (Hocoma; Zurich, Switzerland) as the experimental intervention. Among 10 comparisons, 3 investigated RAGT versus conventional OGT [21, 22, 25] and 2 investigated RAGT versus BWS gait training [17, 18]. Two investigated RAGT versus non-gait-specific training (strength [23] or bike [24]). Finally, three trials compared RAGT with no intervention [16, 19, 20].

The frequency of treatment ranged from three [16, 18–20, 25] to four [23] or five [17, 21, 22] times per week. The duration of treatment was 4 weeks [16, 19, 20, 23, 25], 8 weeks [18, 21, 22] or 12 weeks [17]. One study involved only one treatment session [24]. The treatment intensity (in terms of session duration) ranged from 30 to 60 min and the treatment time did not differ between the control and experimental groups [17, 18, 21–25], with the exception of the no-intervention groups [16, 19, 20]. A summary of the interventions is presented in Table 2.

**Table 2:** Summary of included studies (n=10)

Study	Design	Participants	Intervention	Outcome measures
Alcobendas-Maestro 2012	RCT	*n* = 80 Time since injury (months) = 3 - 6 ASIA scale = C, D Level of injury = C2 to T12 (UMN)	Exp = RAGT 30 min x 5/wk x 6 wk Con = OGT 60 min x 5/wk x 6 wk Both = Usual PT	• Speed = 10-m walk test • Distance = 6-min walk test • Functional level = WISCI II, FIM-L • Leg strength = LEMS • Spasticity = MAS • Pain = VAS • Timing: 0, 8 wk
Duffell 2015	RCT	*n* = 56 Time since injury (months) > 12 ASIA scale = C, D Level of injury = above T10 (UMN)	Exp = RAGT 30~45 min x 3/wk x 4 wk Con = no intervention	• Speed = 10-m walk test • Distance = 6-min walk test • Balance = TUG • Timing: 0, 1, 2, 4 wk
Esclarin-Ruz 2014	RCT	*n* = 88 Time since injury (months) < 6 ASIA scale = C, D Level of injury = C2 to L3 (UMN+LMN)	Exp = RAGT 30 min x 5/wk x 8 wk Con = OGT 30 min x 5/wk x 8 wk Both = Usual PT 60 min x 5/wk x 8 wk	• Speed = 10-m walk test • Distance = 6-min walk test • Functional level = WISCI II, FIM-L • Leg strength = LEMS • Timing: 0, 8 wk
Field-Fote 2011	RCT	*n* = 74 Time since injury (months) > 12 ASIA scale = C, D Level of injury = At or above T10 (UMN)	Exp = RAGT 60 min x 5/wk x 12 wk Con1 = BWS treadmill-based training with manual assistance 60 min x 5/wk x 12 wk Con2 = BWS treadmill-based training with stimulation 60 min x 5/wk x 12 wk Con3 = OGT with stimulation with BWS 60 min x 5/wk x 12 wk	• Speed = 10-m walk test • Distance = 2-min walk test • Leg strength = LEMS • Timing: 0, 12 wk
Hornby 2005	RCT	*n* = 35 Time since injury (months) < 6 (14–180 days) ASIA scale = B, C, D Level of injury = Above T10 (UMN)	Exp = RAGT 30 min x 3/wk x 8 wk Con1 = BWS treadmill-based training with manual assistance 30 min x 3/wk x 8 wk Con2 = OGT with BWS 30 min x 3/wk x 8 wk	• Speed = 10-m walk test • Distance = 6-min walk test • Functional level = WISCI II, FIM-L • Leg strength = LEMS • Spasticity = MAS • Balance = TUG • Timing: 0, 8 wk
Labruyere 2014	RCT Cross-over	*n* = 9 Time since injury (months) > 12 ASIA scale = C, D Level of injury = C4 -T11 (UMN)	Exp = RAGT 45 min x 4/wk x 4 wk Con = Strength 45 min x 4/wk x 4 wk	• Speed = 10-m walk test • Functional level = WISCI II • Leg strength = LEMS • Pain = VAS • Balance = BBS • Timing: 0, 1, 2, 3, 4 wk
Niu 2014	RCT	*n* = 40 Time since injury (yrs) = Exp 8.9±9.9, Con 7.5±5.5 ASIA scale = B, C, D Level of injury = above T10 (UMN)	Exp = RAGT 60 min x 3/wk x 4 wk Con = no intervention	• Speed = 10-m walk test • Distance = 6-min walk test • Balance = TUG • Timing: 0, 1, 2, 4 wk
Shin 2014	RCT	n = 60 Time since injury (months) < 6 ASIA scale = D Level of injury = UMN	Exp = RAGT 40 min x 3/wk x 4 wk Con = OGT 30 min x 3/wk x 4 wk Both = Usual PT 30 min x 2/wk x 4 wk	• Functional level = WISCI II, SCIM3, AMI • Leg strength = LEMS • Timing: 0, 4 wk
Tang 2014	RCT	*n* = 30 Time since injury (months) = 6.3 ASIA scale = D Level of injury = T8 to L3 (UMN+LMN)	Exp = RAGT 40 min Con = Bike 40 min	• Speed = 10-m walk test • Agility = probe reaction time • Timing: before and after the intervention
Varoqui 2014	RCT	*n* = 30 Time since injury (yrs) = Exp 11.80±2.54, Con 8.09±1.89 ASIA scale = C, D Level of injury = above T10 (UMN)	Exp = RAGT 60 min x 3/wk x 4 wk Con = no intervention	• Speed = 10-m walk test • Distance = 6-min walk test • Balance = TUG • Timing: 0, 4 wk

*Exp* experimental group, *Con* control group, *RAGT* robotic-assisted gait training, *OGT* over-ground training), Strength**,** *BWS* body weight-supported gait training, *WISCI* walking index for spinal cord injury, *LEMS* lower extremities motor scale, *FIM-L* Functional Independence Measure – Lokomotion, *MAS* modified Ashworth scale, *VAS* visual analog scale, *TUG* timed up and go, *AMI* ambulatory motor index

### Outcomes

All included studies investigated improvement in ambulatory function measured as speed (m/s, 10 m walk test) [16, 19–24] and capacity of walking (meters walked in 6 min [16, 21, 22] or 2 min [17]) as primary outcomes. Walking aids were allowed in all studies. Other frequently investigated outcomes were: leg strength measured as LEMS (lower extremity motor score of the neurological examination according to the American Spinal Injury Association International Standards [34], range 0 to 50) [17, 18, 21–23, 25], level of functional mobility and independence measured by WISCI-II (assesses the amount of physical assistance needed, as well as devices required) [18, 23, 25],, FIM-L (independence of gait) [18, 21, 22], functional mobility and balance measured by TUG [16, 19, 20], and spasticity measured as MAS [18, 21].

#### Effects on gait velocity

**Fig. 2:**
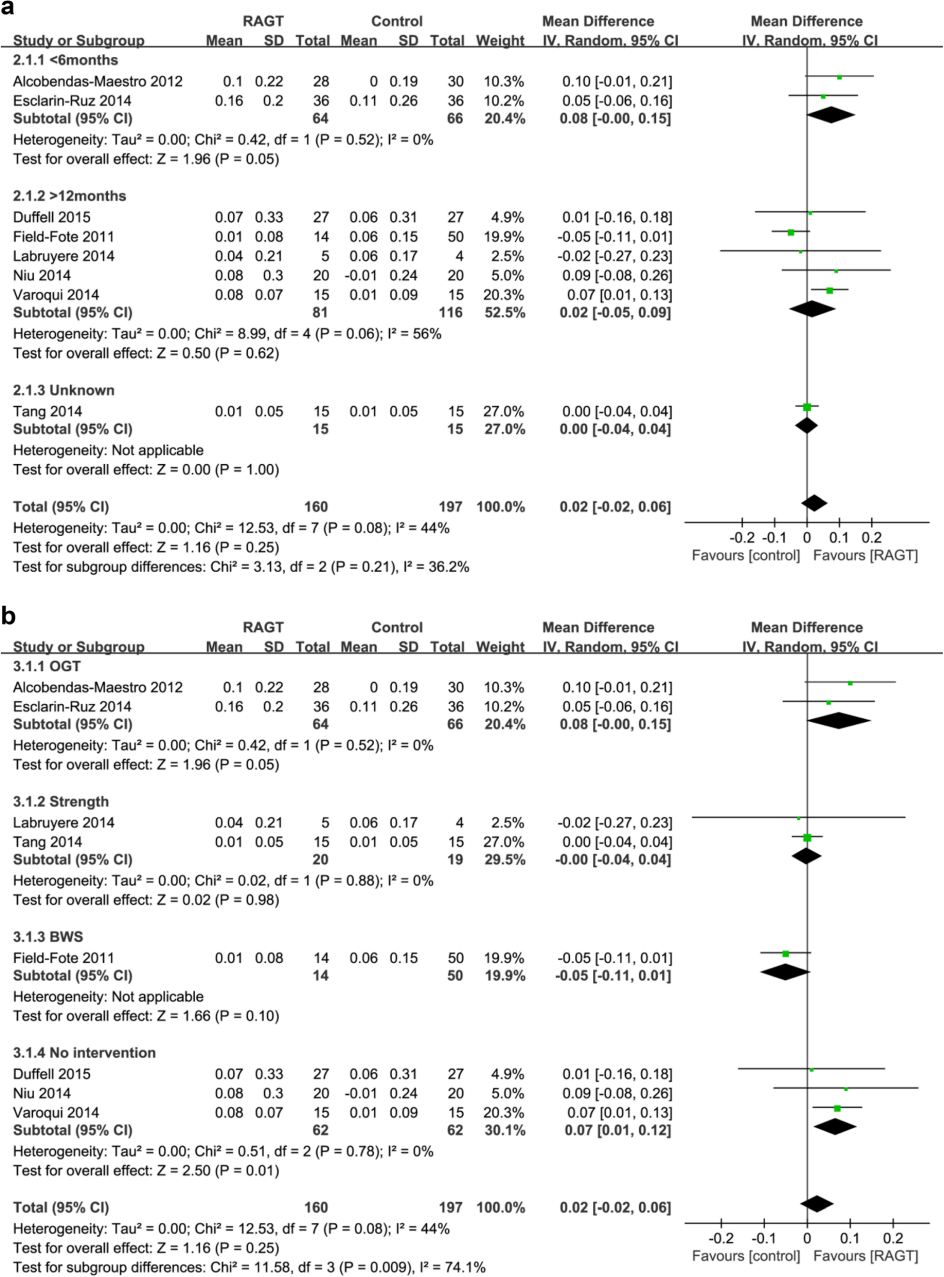
Weighted mean difference (95% CI) of the effect of RAGT compared with control on gait speed by pooling data from 8 trials (*n* = 355) with subgroup analysis by (**a**) time since injury (acute < 6 months, chronic > 12 months) and (**b**) type of intervention (BWS, OGT, strength and no intervention) in people with SCI

Gait velocity tended to be higher in the acute RAGT groups than in the OGT groups, albeit not significantly so (pooled mean difference = 0.08 m/s, 95% CI -0.00 to 0.15; P = 0.05; I^2^ = 0%, two trials, 130 participants) (Fig. 2). In the chronic RAGT groups, significantly greater improvements were observed than in the no intervention groups (pooled mean difference = 0.07 m/s, 95% CI 0.01 to 0.12, *P* = 0.01, I^2^ = 0%; three trials, 124 participants).

#### Effects on gait distance

**Fig. 3:**
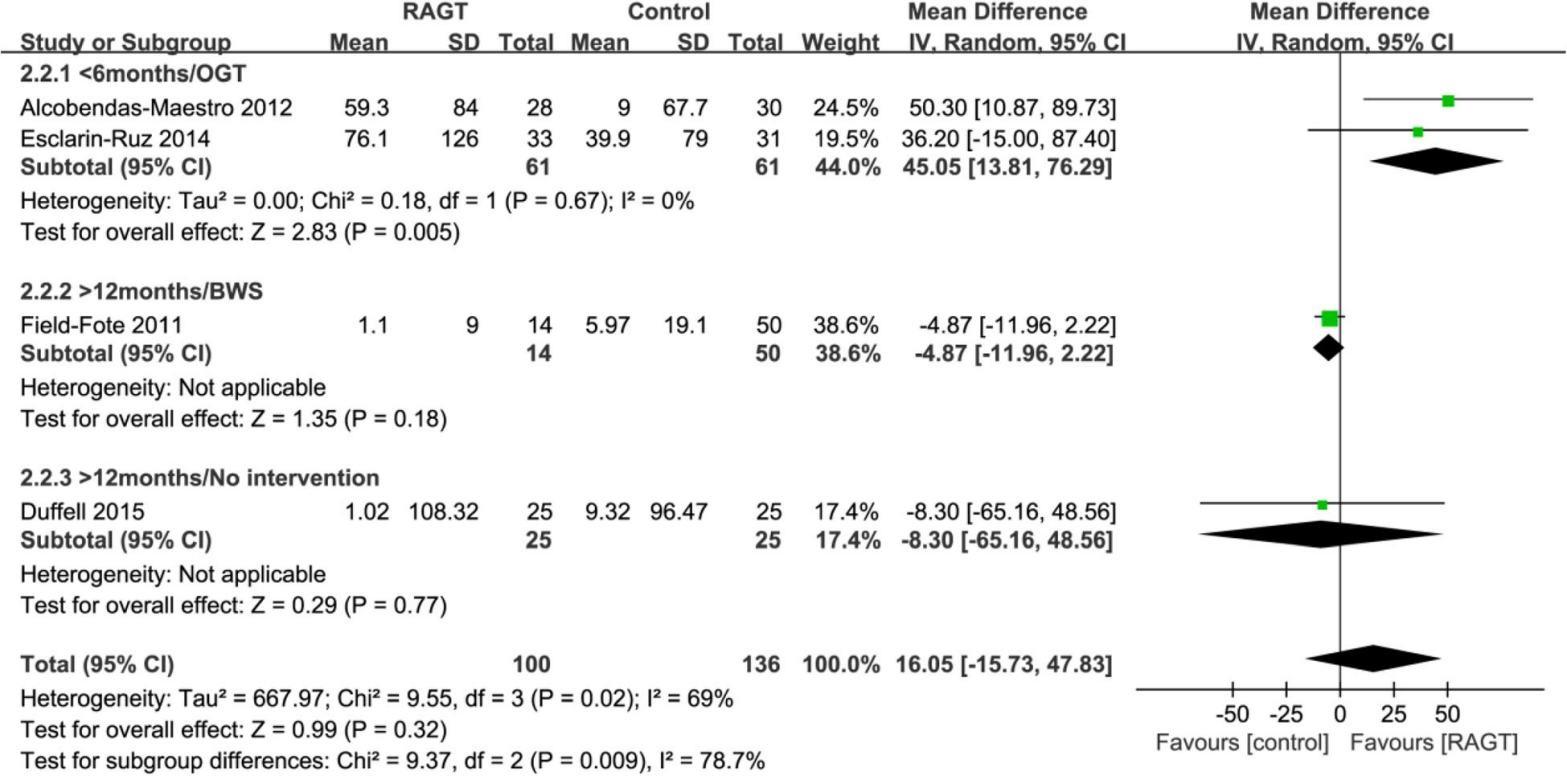
Weighted mean difference (95% CI) of the effect of RAGT compared with control on gait distance by pooling data from 4 trials (*n* = 298) with subgroup analysis by time since injury (acute < 6 months, chronic > 12 months) and type of intervention (BWS, OGT, and no intervention) in people with SCI

Significantly greater improvements were observed in the acute RAGT groups than in the OGT groups (pooled mean difference = 45.05 m, 95% CI 13.81 to 76.29; *P* = 0.005; I^2^ = 0%, two trials, 122 participants) (Fig. 3). However, there were no significant improvements in the chronic RAGT groups compared to the BWS or no-intervention groups (pooled mean difference = -4.92 m, 95% CI -11.96 to 2.11; P = 0.17; I^2^ = 0%, two trials, 114 participants).

#### Effects on leg strength

**Fig. 4:**
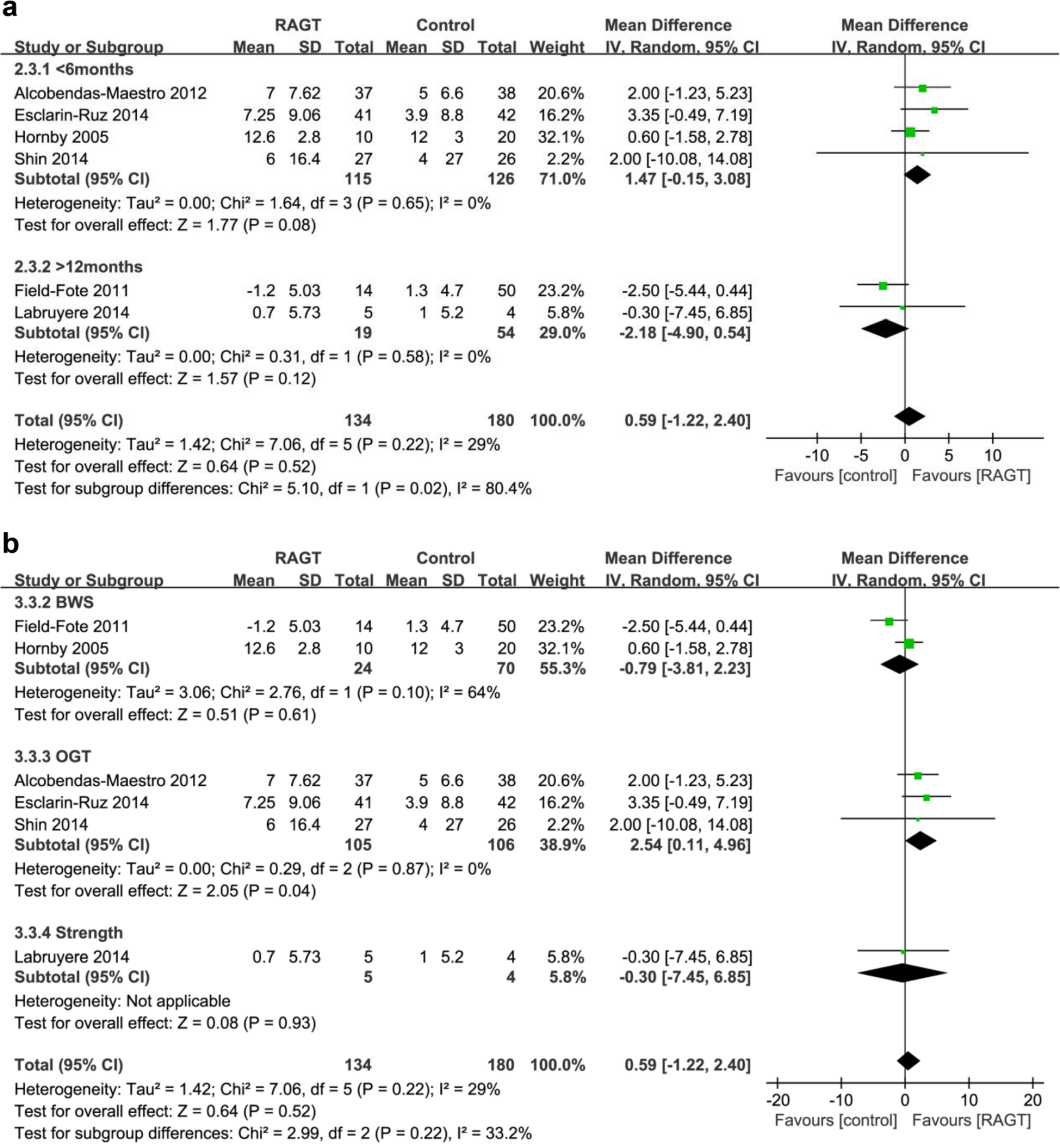
Weighted mean difference (95% CI) of the effect of RAGT compared with control on leg strength (LEMS) by pooling data from 6 trials (*n* = 314) with subgroup analysis by (**a**) time since injury (acute < 6 months, chronic > 12 months) and (**b**) type of intervention (BWS, OGT, and strength) in people with SCI

In the acute RAGT, leg strength measured by LEMS was greater than that in the OGT groups (pooled mean difference = 2.54, 95% CI 0.11 to 4.96, P = 0.04, I^2^ = 0%; three studies, 211 participants) (Fig. 4). However, there was no significant improvement in the chronic RAGT groups compared to the BWS and strength groups (pooled mean difference = -2.18, 95% CI -4.90 to 0.54; P = 0.12; I^2^ = 0%, two studies, 73 participants).

#### Effects on functional level of mobility and independence

**Fig. 5:**
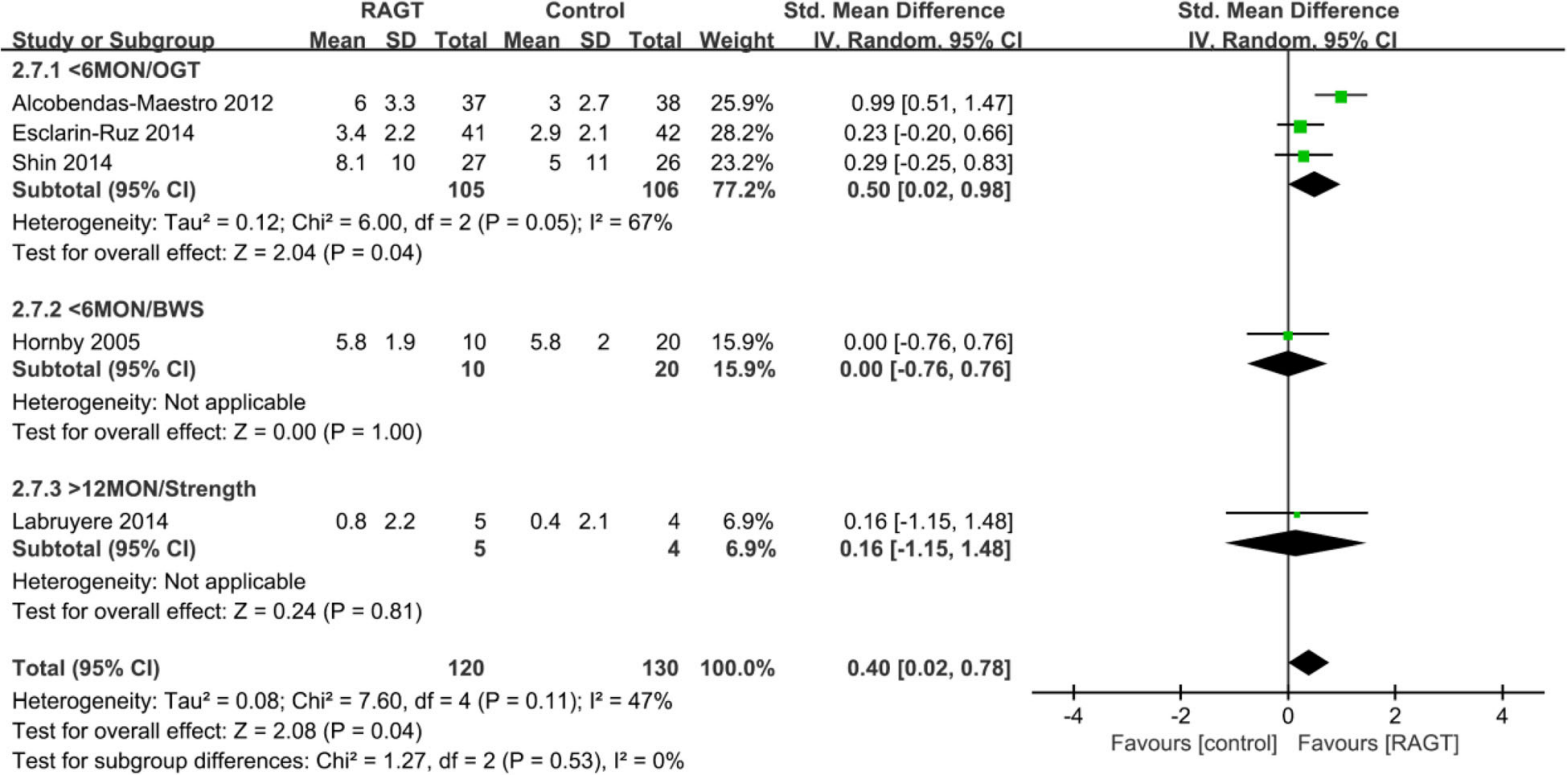
Weighted mean difference (95% CI) of the effect of RAGT compared with control on functional level and independence (WISCI-II and FIM-L) by pooling data from 5 trials (*n* = 250) with subgroup analysis by time since injury (acute < 6 months, chronic > 12 months) and type of intervention (BWS, OGT and strength) in people with SCI

Significantly greater improvements on the WISCI-II and FIM-L were observed in the acute RAGT groups compared to the OGT groups (pooled mean difference = 0.5, 95% CI 0.02 to 0.98; P = 0.04; I^2^ = 67%, three trials, 211 participants) (Fig. 5). There was no significant improvement in the chronic RAGT groups compared to the strength group (mean difference = 0.16, 95% CI -1.15 to 1.48, P = 0.81; one trial, 9 participants).

#### Effects on balance

**Fig. 6:**
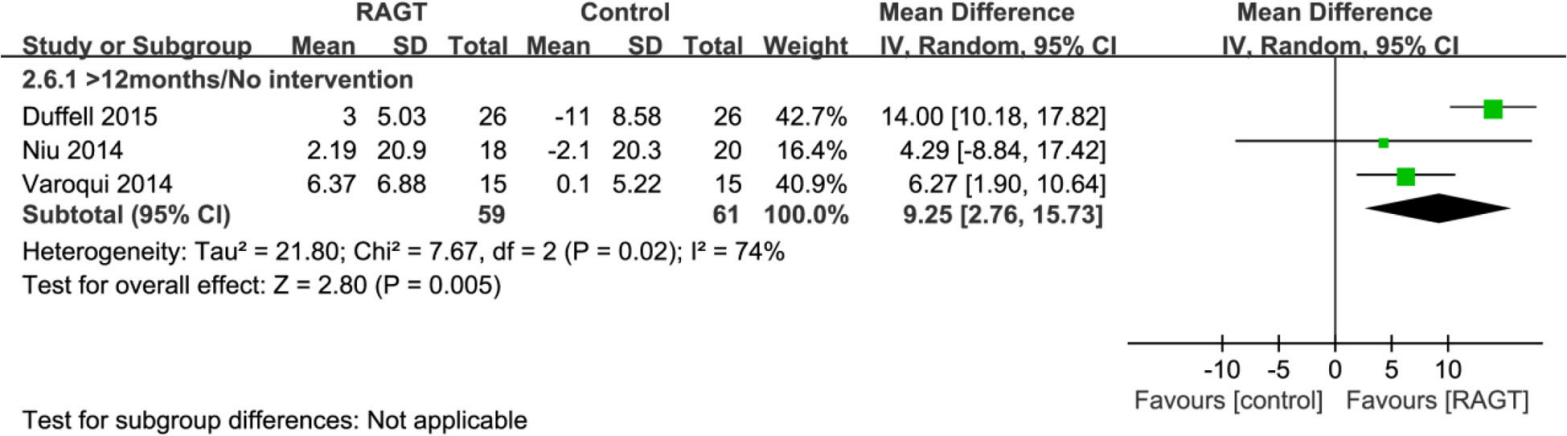
Weighted mean difference (95% CI) of the effect of RAGT on balance (TUG) compared to that in the no intervention group by pooling data from 3 trials (*n* = 125) in people with chronic SCI

Significantly greater improvements in TUG were observed in the chronic RAGT groups compared to the no intervention groups (pooled mean difference = 9.25, 95% CI 2.76 to 15.73, P = 0.005, I^2^ = 74%; three trials, 120 participants) (Fig. 6). No trial with acute participants measured recovery of balance.

#### Effects on spasticity

**Fig. 7:**
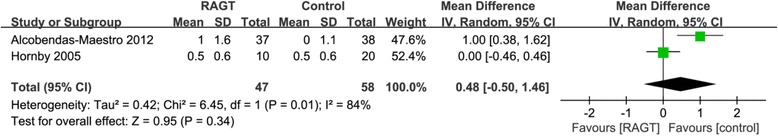
Weighted mean difference (95% CI) of the effect of RAGT on spasticity (MAS) compared to that with control by pooling data from 2 trials (*n* = 105) in people with SCI

The overall changes in spasticity were similar in the control and acute RAGT groups (pooled mean difference was 0.48, 95% CI -0.50 to 1.46, P = 0.34, I^2^ = 84%; 2 trials, 105 participants) (Fig. 7). No trial with chronic participants measured changes in spasticity.

## Discussion

This systematic review aimed to provide an overview of the current evidence on the RAGT approach to gait rehabilitation after incomplete SCI. There was no superiority of the control group in all outcomes. The RAGT group was superior or equivalent when compared to the control group. The data for participants < 6 months post-injury showed improvements in walking distance, lower limb strength and functional level of mobility and independence for RAGT over conventional OGT. The data for participants > 1 year post-injury showed improvements in gait speed and balance with RAGT compared with no intervention. However, there was no greater improvement in walking speed in the acute RAGT groups compared to OGT, and no greater improvement in gait distance, leg strength or functional level of mobility and independence in the chronic RAGT groups compared to the groups that underwnt other forms of physiotherapy.

Studies in spinalized animals have shown that motor potential can be elicited by reciprocal passive movement of the lower limbs [35]. The suggested mechanism is the activation of gait centers in the spinal cord, the CPG. Indeed, similar locomotor activity can be activated in patients with severe spinal cord injuries via passive activation of the legs on a treadmill [36]. Synchronous reciprocal movements of both legs, simulating normal walking, are required to activate the locomotor centers in the spinal cord. Often, two physiotherapists are required to perform these reciprocal movements, which should be highly symmetrical and physiological to stimulate the locomotor centers effectively. It is important to achieve symmetrical and physiological walking with body weight support to accurately stimulate the locomotor centers and therefore activate paralyzed muscles [37].

To improve over-ground walking ability, locomotor therapies that combine a BWS system with a treadmill have been developed over the past two decades [38]. BWS training has been shown to be effective in improving ambulatory function after SCI, although it is not more effective than equivalent over-ground mobility training [29, 39]. A recent meta-analysis of gait training in SCI revealed that the pooled mean between-group (comparing treadmill training with OGT) difference for gait velocity was−0.01 m/s (95% CI−0.09 to 0.08) [39]. These results are equivalent to those of a 2012 Cochrane review [40]. Another strategy to improve walking after SCI is to administer BWS treadmill training combined with functional electrical stimulation (FES). Its benefits have been noted in terms of CNS regeneration, initiation of stepping, improving foot clearance, knee extension, and strength in both acute and chronic participants [41–43]. However, a recent meta-analysis revealed that people with SCI who used FES did not significantly increase their walking speed and capacity when compared with patients who were treated with other approaches [40].

RAGT has many advantages over conventional BWS treadmill training methods, including early initiation of gait training in severely dependent patients, less effort for physiotherapists, longer duration and higher intensity of gait, more physiological and reproducible gait patterns, and the possibility to measure a patient’s performance [44]. These factors contribute to spinal and central neuroplasticity. RAGT allows wheelchair-bound patients to practice up to 1000 steps during a 30 min session, compared with a maximum of only 50–100 steps during a conventional therapy session [45]. Furthermore, according to recent studies, RAGT has potential aerobic benefits and a positive influence on cardiopulmonary fitness in severely disabled spinal cord and stroke patients [46]. Also, RAGT may have some positive effect on other parameters associated with quality of life in SCI patients, including cardiopulmonary function, regulation of bowel movements, and bone density [47, 48].

Three types of robotic-assisted device have been developed: exoskeleton type, end-effector type and portable powered robotic exoskeletons [49–51]. Examples of end-effector devices are the “G-EO-System” [50], the “Lokohelp” [52], the “Haptic Walker” [53], and the “Gait Trainer GT 1” [54]. The definition of an end-effector principle is that a patient’s feet are placed on footplates whose trajectories simulate the stance and swing phases during treadmill gait training [50]. The two prototypes of the exoskeleton type are the “LOPES” (University of Twente, Netherlands) [53] and “Lokomat” (Hocoma; Zurich, Switzerland) [37], a treadmill-based walking machine comprised of a harness which carries patients in an upright position and robotic arms attached to the patient’s legs and allows physiological and symmetrical reciprocal movement on a treadmill. Compared to treadmill-based gait orthoses, portable powered robotic exoskeletons are compact and wearable, they provide individuals with complete paralysis the ability to walk independently over-ground in a natural reciprocal pattern. Only 10 RCTs of the exoskeleton type (all involving the Lokomat) have been performed to date, and no RCT of the end-effector type or portable powered exoskeletons has been carried out.

Some reviews have assessed the quality of current evidence on the effectiveness of RAGT in spinal cord injury patients, focusing on walking ability and performance, but no evidence that RAGT improves walking function more than other locomotor training strategies has been reported [55]. In particular, Harvey [39] showed that there is nothing intrinsically therapeutic about cyclic walking with robotic devices, although RAGT may provide a convenient and safe way for therapists to provide intensive repetitive practice. Also, Dobkin [56] suggested in a scientifically-conducted efficacy trial that RAGT should not be routinely provided to disabled, vulnerable persons. However, this meta-analysis showed that participants < 6 months post-injury demonstrated improvements in walking distance, functional level of mobility and independence and lower limb strength with RAGT over conventional OGT intervention. The evidence for the mechanisms underlying functional improvements in humans is poor, particularly in terms of neural changes in the spinal cord. However, RAGT has been shown to lead to changes in many spinal reflex pathways behave more like those of healthy controls following robot-mediated training in patients with SCI [57]. Of particular importance is the finding that, among other changes to the spinal reflex circuitries, robotic-assisted step training in SCI patients resulted in the re-emergence of a physiological phase modulation of the soleus H-reflex during walking [58].

The role of RAGT in gait rehabilitation is similar to that in other central nervous system disorders such as stroke, traumatic brain injury, and multiple sclerosis. A recent meta-analysis of 23 trials with a total of 999 stroke patients showed a small additional value of RAGT combined with conventional training compared to conventional training alone, especially for acute patients. In traumatic brain injury patients, a recent systematic review showed that RAGT may have a beneficial effect on the rehabilitation process and is feasible and effective in improving gait function in multiple sclerosis patients [44].

A novel cable-driven robotic gait training system can provide controlled forces to the limb during the swing phase of gait to produce an optimal training paradigm with either assistance or resistance [59]. One pilot RCT study showed that cable-driven robotic resistance training can be used as an adjunct to BWS treadmill training to improve overground walking function in humans with chronic incomplete SCI compared to assistance training [60]. Another pilot RCT study provided evidence that Lokomat-applied resistance training may improve performance in skilled overground walking tasks in patients with chronic motor incomplete SCI compared to conventional Lokomat-assisted gait training [61]. Greater cognitive engagement during training may have elicited greater involvement of cortical regions associated with gait, which are particularly involved in the adjustments of motor output during swing [62], and cable-driven robotic resistance training is promising gait training for chronic incomplete SCI patients.

No study has yet been published that directly compares different types of devices, end-effector, or exoskeleton devices. Furthermore, no data are available regarding the optimal RAGT protocol. Larger controlled studies are required to determine the optimal timing and protocol design that will maximize efficacy and long-term outcomes in neurological patients. Another important issue is the availability and cost of these devices. At present, their usage is limited to highly specialized centers with the required space and resources. Therefore, more compact and affordable devices for home use are needed [44]. In addition, some technical limitations have been identified. The fixed trajectory control strategy used in both types of robotic systems may encourage passive rather than active training and may eliminate the variability in kinematics of the lower limbs, which is thought to be critical for successful motor adaptation. The limited degree of freedom in the exoskeleton type, which allows movement only in the sagittal plane, may limit the natural walking pattern and affect gait dynamics.

The methodologic quality of all included trials was either low or good, and the study designs differed considerably. Moreover, eligibility criteria, randomization processes, timing of treatment, intervention parameters, and their execution, frequency, and intensity were commonly heterogeneous or not clearly stated in the articles [2]. Failure to comply with the high methodologic requirements of RCTs significantly increases the risk of bias within these trials, as represented in the final PEDro scores. The power of the findings and their implication for clinical practice are thereby diminished [63].

## Conclusions

This review provides evidence that the acute RAGT group showed significantly greater improvements in gait distance, strength, and functional level of mobility and independence than the OGT group. In the chronic RAGT group, significantly greater improvements in speed and balance were observed than in the group with no intervention.

RAGT treatment in incomplete SCI patients showed promise in restoring functional walking. An improvement in locomotor ability in persons with SCI using RAGT might enable them to maintain a healthy lifestyle and increase their level of physical activity.

## Abbreviations

BWS: Body weight-supported gait training
CI: Confidence intervals
CPG: Central pattern generators
FES: Functional electrical stimulation
FIM-L: Functional independence measure – locomotion
LEMS: Lower extremities motor scale
MAS: Modified Ashworth scale
OGT: Over-ground training
RAGT: Robot-assisted gait training
RCTs: Randomized controlled trials
SCI: Spinal cord injury
TUG: Timed up and go
WISCI: Walking index for spinal cord injury
